# Supplementary material for: Associations between Pharmacotherapy for Cardiovascular Diseases and Periodontitis
Source: Int J Environ Res Public Health. 2021 Jan 18;18(2):770. doi: 10.3390/ijerph18020770 (PMC7831110; doi:10.3390/ijerph18020770)
Supplement: Supplementary file 1 [file ijerph-18-00770-s001.pdf]

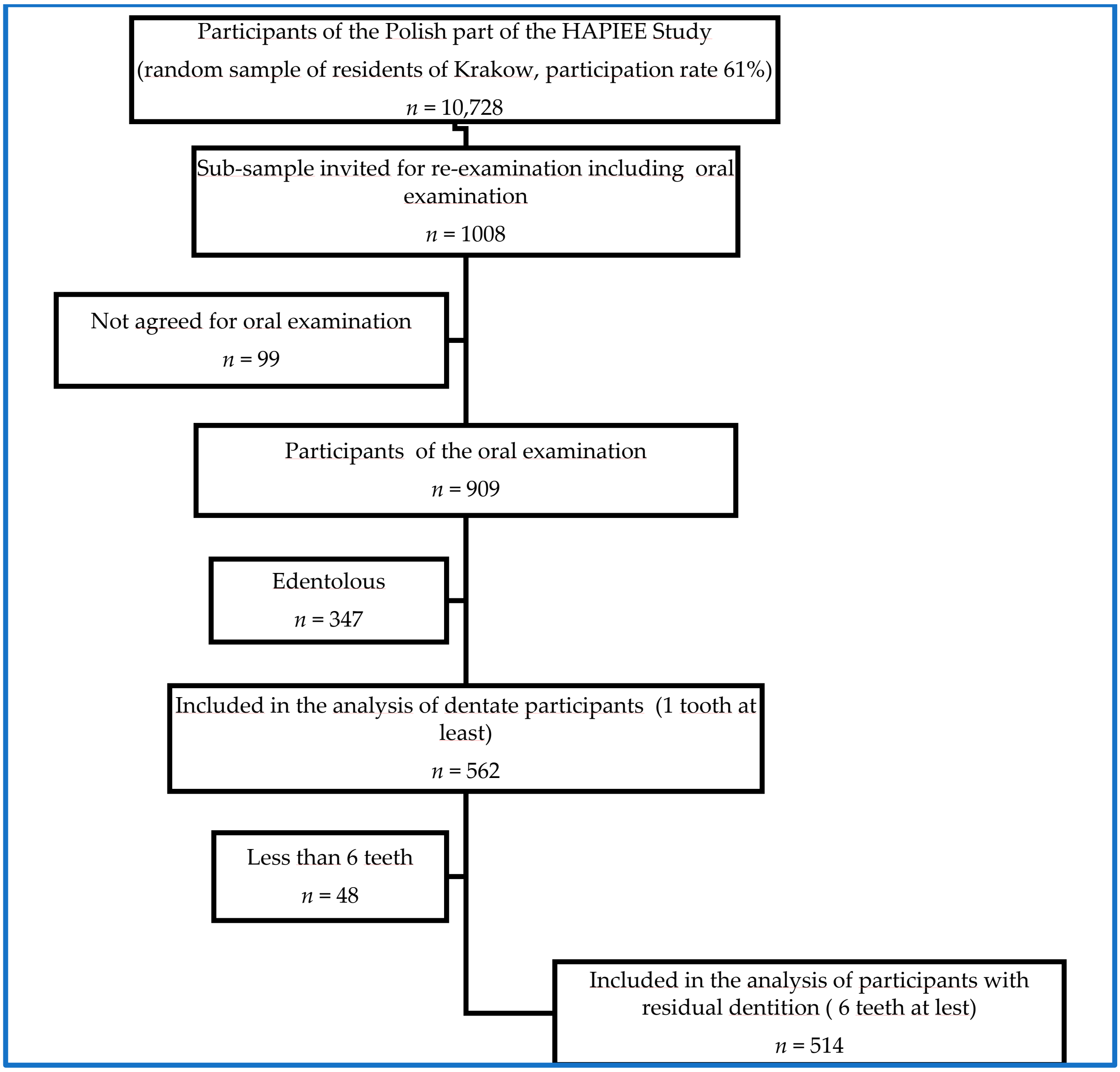

Figure S1. Sample selection, recruitment and restrictions (HAPIEE = Health Alcohol and Psychosocial factors in the Eastern Europe)
